# Evaluation of meal supervision for patients attending an eating disorders day program

**DOI:** 10.1186/2050-2974-2-S1-O22

**Published:** 2014-11-24

**Authors:** Lisa Herbert, Jessica Wheatley, Susan Hart

**Affiliations:** 1 Derwent House Day Program, Sydney, Australia

## Background

Supervision of meals is an essential part of intensive eating disorder treatment. However, there is a lack of evidence regarding the most effective approach when delivering this service. This study evaluated an eating disorder day program's core components of meal supervision against set criteria, and how meal supervision was received by patients.

## Methods

All staff of an eating disorders day program were observed completing meal supervision by an external assessor over a three week period. An audit tool was developed using the program's established mealtime guidelines. Descriptive feedback was sought to evaluate the patients' experience.

## Results

One hundred percent of meals assessed were completed within program guidelines and done so consistently by all staff. Staff commonly used time and behaviour prompts. They explained meal guidelines inconsistently, but as deemed necessary. Staff were more confident with food than fluid guidelines. Patients valued autonomy and less support as they progressed in treatment.

## Conclusion

Day program staff were compliant with the program's mealtime guidelines and were consistent when doing so. Meal supervision was regarded as a positive aspect of treatment, and patients valued meal support being tapered as they advanced.

This abstract was presented in the **Service Initiatives** stream of the 2014 ANZAED Conference.

